# Antimicrobial Resistance Pattern, Clustering Mechanisms and Correlation Matrix of Drug-Resistant *Escherichia coli* in Black Bengal Goats in West Bengal, India

**DOI:** 10.3390/antibiotics11101344

**Published:** 2022-10-01

**Authors:** Jaydeep Banerjee, Debaraj Bhattacharyya, Md Habib, Siddharth Chaudhary, Suman Biswas, Chinmoy Maji, Pramod Kumar Nanda, Arun K. Das, Premanshu Dandapat, Indranil Samanta, Jose M. Lorenzo, Triveni Dutt, Samiran Bandyopadhyay

**Affiliations:** 1 ICAR-Indian Veterinary Research Institute, Eastern Regional Station, 37 Belgachia Road, Kolkata 700 037, India; 2 Faculty of Veterinary Science, West Bengal University of Animal and Fishery Sciences, Kolkata 700 037, India; 3 Centro Tecnológico de la Carne de Galicia, Avd. Galicia nº 4, Parque Tecnológico de Galicia, San Cibrao das Viñas, 32900 Ourense, Spain; 4 Área de Tecnoloxía dos Alimentos, Facultade de Ciencias, Universidade de Vigo, 32004 Ourense, Spain; 5 Division of Livestock Production and Management, ICAR-Indian Veterinary Research Institute, Izatnagar 243 122, India

**Keywords:** *acrAB*, AMR, carbapenem, diarrhoea, ESBL, farming practice, goat

## Abstract

A cross-sectional study covering four agro-climatic zones of West Bengal, India, was carried out to understand the risk-factors, antimicrobial resistance mechanism and clustering of the resistance characteristics of *Escherichia coli* isolated from healthy (170) and diarrhoeic (74) goats reared under intensive (52) and semi-intensive (192) farming practices. Of the 488 *E. coli* isolates, the majority, including the extended spectrum (n: 64, 13.11%) and AmpC β-lactamase (ACBL) (n: 86, 17.62%) producers, were resistant to tetracycline (25.2%), followed by enrofloxacin (24.5%), cefotaxime (21.5%) and amikacin (20.5%). Statistical modelling revealed that the isolates from diarrhoeic animals (*p* < 0.001) are likely to be more ACBL-positive than those from the healthy counterparts. Similarly, cefotaxime (*p* < 0.05) and enrofloxacin-resistance (*p* < 0.01) were significantly higher in diarrhoeic goats and in goats reared intensively. The isolates (n = 35) resistant to multiple drugs revealed the presence of β-lactamase [*bla*CTXM-1-(21), *bla*SHV-(7), *bla*TEM-(3), *bla*CMY-6-(1), *bla*CITM-(3)]; quinolone [*qnr*B-(10), *qnr*S-(7), *aac*(6’)-*Ib-cr-(*3)]; tetracycline [*tet*A-(19), *tet*B-(4)] and sulphonamide resistance determinants [*sul*1-(4)]; multiple plasmids, especially those belonging to the IncF and IncI1 replicon types; and active *acrAB* efflux pumps. Further, two isolates harbored the carbapenem resistance (*bla*NDM-5) gene and eight were strong biofilm producers. This first ever study conducted to unravel the status of AMR in goat farming reveals that not only the intensive farming practices but also certain clinical ailments such as diarrhoea can increase the shedding of the drug-resistant isolate. The emergence of multi-drug resistant (MDR) *E. coli* in goats, particularly those that are carbapenem resistant, is a cause for concern that indicates the spread of such pathogens even in the livestock sub-sector generally considered as naive.

## 1. Introduction

The rearing of goats often requires less start-up capital but offers enormous potential towards increasing the cash income of resource-poor and marginalized farming communities in the Indian sub-continent [1]. The meat of the Black Bengal goat is preferred because of its delicious taste and there is a great demand for its skin/hide and fiber in the international market. Although the majority rear this breed via semi-intensive farming in the backyard sector, the practice of intensive rearing is gaining momentum with new risks and threats [2] including the increased prophylactic or metaphylactic use of antimicrobials (AMU) leading to the emergence and spread of antimicrobial resistance (AMR), possibly the most dreaded threat ever faced by mankind [3]. 

Limited epidemiological studies in different parts of the world have revealed that goats, similar to other food animals, can act not only as a mere reservoir but also a potential spreader of the AMR pathogens or antibiotic resistance gene (ARG) through its milk and meat [4,5]. AMR pathogens such as ESBL and carbapenemase producers as well as methicillin-resistant and vancomycin-intermediate *Staphylococcus aureus* have been reported in different food and companion animals, including goats [6,7,8]. Altogether, the recent observations illustrate such a radical shift in perspective that goat farming can no longer be considered a farming system in isolation that presents no or minimum loads of AMR. Recently, we reported a higher proportion of resistant pathogens in cattle with diarrhoea than their healthy counterparts [8]. Diarrhoea, a multifactorial condition, is not uncommon in goats and often requires antimicrobial or antiparasitic intervention. However, no comprehensive study is yet available to reflect the status of AMR commensals or ARGs in goats. Further, the effect of farming practices, health condition and agro-climatic factors on the prevalence of AMR pathogens has not yet been investigated.

Many complex mechanisms are associated with the emergence of plasmid-mediated antibiotic resistance in microorganisms; notable among these pathogens are ESBLs, metallo β-lactamases (MBL) and plasmid-mediated quinolone resistance (PMQR) genes [9,10]. In addition, active efflux pumps—an important mechanism involved in biofilm formation [11]—can also be employed by these pathogens to enhance drug-resistance as a part of their survival strategy in adverse conditions. Among various pathogens of interest, *Escherichia coli*, because of their ubiquitous presence and propensity to acquire and preserve resistance characteristics, are often preferred as indicators to assess the real-time trends of the prevalence of AMR in any environment [12]. Therefore, this study explores the AMR profile of caprine *E. coli* isolates from different agro-climatic zones of West Bengal (India) and assesses the risk of resistance acquisition against the health condition of goats (diarrhoea vs. non-diarrhoea), the farming practices (intensive vs. semi-intensive) and the agroclimatic zones. Further, the study describes the resistance characteristics of MDR *E. coli* isolated from goats.

## 2. Results

### 2.1. Drug Resistance Characteristics of the Caprine E. coli Isolates

All the rectal swabs were positive for *E. coli* and, altogether, we screened 488 *E. coli* isolates. Of these, ESBL and ACBL production were confirmed in 64 [13.11% (10.31–16.51)] and 86 [17.62% (14.41–21.36)] isolates, respectively. The overall resistance profile showed that *E. coli* isolates were frequently resistant to enrofloxacin [24.5%; 95% CI (20–28)], tetracycline [25.2%; 95% CI (21–29)], cefotaxime [21.5%; 95% CI (18–25)] and gentamicin [20.5%; 95% CI (17–24)]. However, they were least resistant to cotrimoxazole [(9.8%; 95% CI (7–12)], amoxicillin-clavulanic acid [12%; 95% CI (9–15)], imipenem [10.3%; 95% CI (8–13)] and chloramphenicol [11.7%; 95% CI (9–15)] (Figure S2). The proportion for each resistance characteristic with respect to each risk factor was categorized as per the guidelines provided by the European Food Safety Authority (https://multimedia.efsa.europa.eu/dataviz-2017/index.htm, accessed on 12 September 2022) and described in Table 1. The resistance levels were found to be moderate except for ampicillin, amikacin, tetracycline, enrofloxacin and cefotaxime, where a high level was found (>20% to 50%). The ACBL positivity was moderate (<20 %) in all the categories, except in the animals with diarrhoea (high (>20%)). In general, imipenem resistance was found to range between the low to moderate level. About 26% of the samples yielded isolates with considerable difference in their susceptibility profiles.

### 2.2. Clustering of Antibiotics and β-Lactam Resistance Mechanism and Their Correlation Matrix

**Figure 1:**
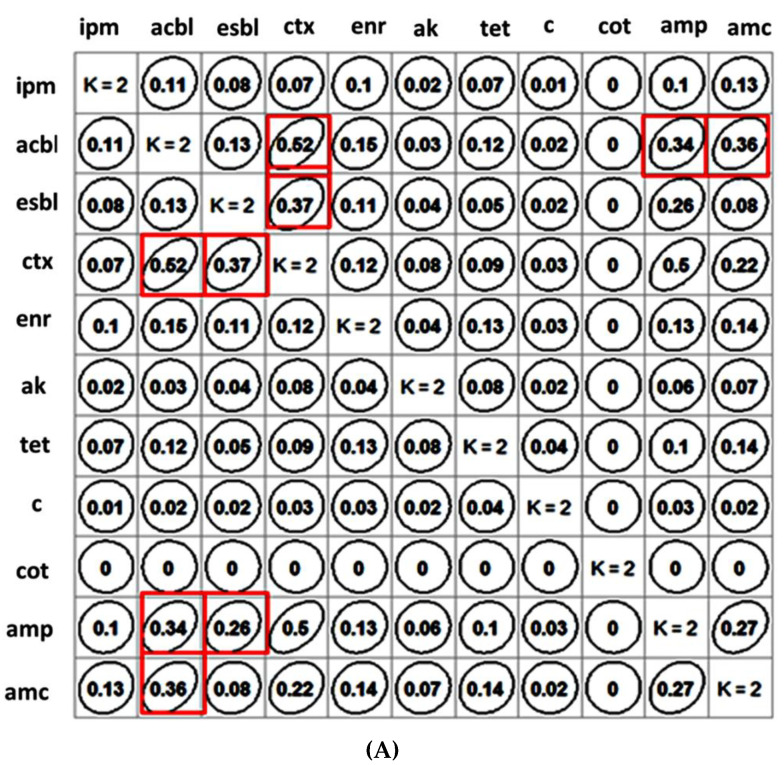

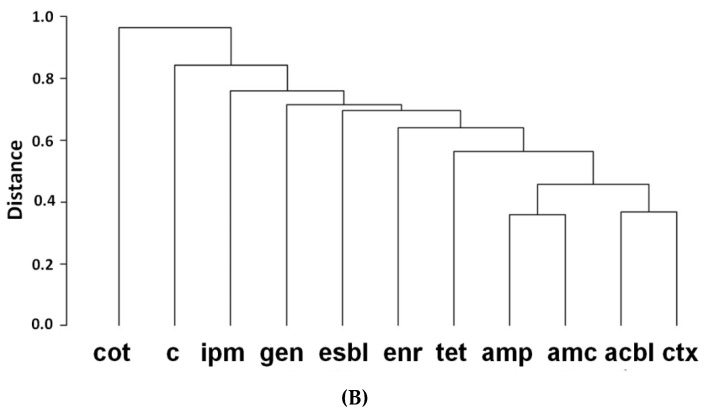
(**A**) Association matrix reflecting the strength and direction of the relationships between individual antimicrobials based on the resistance profile of the *Escherichia coli* isolates investigated in this study. Goodman-Kruskal tau association was used to depict the association matrix highlighting only the antibiotics having statistically significant (*p* < 0.05) association with ACBL and ESBL in red frame. **(B)** Hierarchical tree of nine antibiotics and β-lactam-resistant mechanisms (ESBL and ACBL) based on the resistance profile of the *Escherichia coli* isolates investigated in this study using the cluster analysis derived from UPGMA agglomeration method and dissimilarity structure produced by Euclidean distance matrix (COT—cotrimoxazole; C—chloramphenicol; IPM—imipenem; GEN—gentamicin; ESBL—extended spectrum β-lactamase; ENR—enrofloxacin, TET—tetracycline; AMP—ampicillin; AMC—amoxyclav; ACBL—AmpC-type β-lactamase; CTX—cefotaxime).

The Goodman and Kruskal’s association matrix (Figure 1A) and the cluster analysis (Figure 1B) depicts the concurrent resistance/susceptibility between each pair of antibiotics and the occurrence of β-lactamases (ESBL and ACBL) among all the *E. coli* isolates investigated in this study. It can be deduced from the association matrix (Figure 1A) that most of the ACBL producers were cefotaxime- and amoxicillin-clavulanic acid-resistant and thus clustered together (Figure 1B). The association measure of cefotaxime (0.52) and amoxicillin-clavulanic acid (0.36) resistance with ACBL was higher than that with ESBL (0.36:0.26).

### 2.3. Logistic Regression Analysis

The odds ratios for the occurrence of ESBL, ACBL and resistance to all the antimicrobials tested were calculated in the intensive farms against semi-intensive farms, in diarrhoeic animals against healthy animals and in all three agroclimatic zones against an old alluvial zone (Table 1). We could not find any association of diarrhoea, the applied farming practice and the agro-climatic zones with the resistance characteristics of the caprine isolates, except with ACBL positivity and cefotaxime and enrofloxacin resistance. The caprine isolates from diarrhoeic animals were likely to be more ACBL positive than those from the healthy animals [OR: 1.9 (1.2–3.0), *p* < 0.001]. The effect of the farming practice and the agro-climatic zones remains insignificant. The cefotaxime resistance of the isolates from intensive farms (28.6%) was significantly higher [OR: 1.50 (0.97–2.31), *p* < 0.05] than that from semi-intensive farms (21%). Again, diarrhoeic animals (28%) were found to be significantly [OR: 1.5 (0.94–2.38), *p* < 0.05] more frequently colonized with cefotaxime-resistant populations than their healthy counterpart (20.7%). A similar trend was observed regarding enrofloxacin resistance, which was found to be significantly higher among the isolates from diarrhoeic animals [OR: 1.6 (1.1–2.5), *p* < 0.01] and from the goats under intensive farming [OR: 1.7 (1.0–2.6), *p* < 0.01] than their respective counterparts. 

### 2.4. Molecular Characterization of the MDR E. coli Isolates and Plasmid Incompatibility Types 

In total, 35 *E. coli* isolates were found to be MDR (Table 2). Of them, 20 isolates exhibited ESBL in a standard phenotypic assay, 25 presented ACBL and 15 isolates were both ESBL and ACBL producers. Eight (8) isolates were strong biofilm producers. Most of the isolates were found to possess *bla*CTXM-1 (21) (MZ254711, MZ275237), followed by *bla*AmpC (13) (OL554872), *bla*SHV (7) (MZ254710) and *bla*TEM (3) (MZ254713) genes. Plasmid-mediated cephalosporinase, *bla*CMY-6 (1) (MZ254707) and CITM (3) (MZ275241) were detected in a few isolates. Other resistance determinants included PMQR [*qnr*B (10) (OL554873), *qnr*S (7) (OL554874), *aac(6′)-Ib-cr* (3) (MZ275235)], tetracycline resistance genes [*tet*A (19) (MZ275242), *tet*B (4) (MZ254709)] and sulphonamide resistance genes [*sul*1 (4) (MZ275239)]. Two isolates harboured the *bla*NDM-5 gene (OL554875). The majority of the caprine MDR *E. coli* isolates revealed more than one plasmid type with a predominance of IncF (FIA, FIB and FrepB) and IncI1.

None of the drug-resistant isolates were positive for shiga-toxin or enterotoxigenic virulence factors. However, a few possessed uropathogenic genes such as *iucD* and *papC*. While three carried both *iucD* and *papC*, seven had *iucD* alone and a single isolate had *papC* alone. In total, *iucD* was present in 10 isolates, of which 7 were from diarrhoeic goats and 3 from healthy ones. All the *papC* positive isolates were from goats with diarrhoea.

### 2.5. Efflux Pump Mediated Resistance 

**Figure 2:**
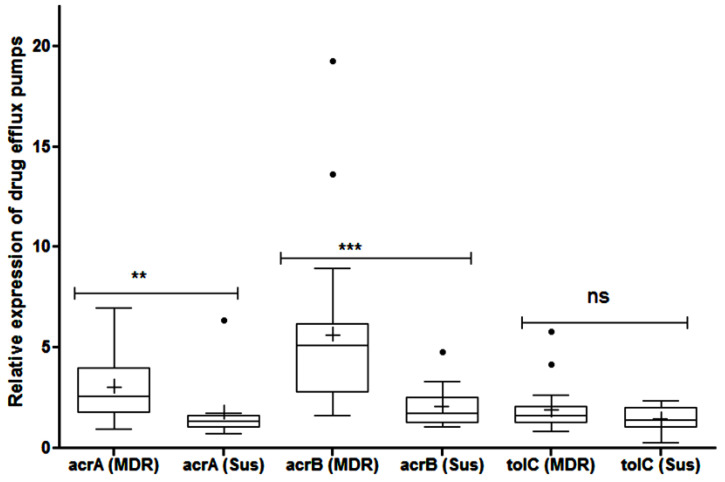
Relative expression of efflux pump system (*acr*A, *acr*B and *tol*C) in multi-drug resistant and pan-susceptible *Escherichia coli* isolates of caprine origin as determined by qPCR with respect to its housekeeping gene *rps*L. Each group is represented by median (-), mean (+) and 95% confidence interval. ns—non-significant, ** (*p* < 0.01), *** (*p* < 0.001) and ● (outliers).

The role of an efflux pump for fluoroquinolone and carbapenem resistance was evident in 10 and 3 isolates, respectively. Compared to the housekeeping gene *rps*L, the relative median expressions of *acr*A and *acr*B among the MDR strains were 2.62 (95% CI: 2.3–5.8) and 5.13 (95% CI: 4–7.2), respectively, which were significantly (*p* < 0.01) higher than the corresponding values [*acr*A: 1.33 (95% CI: 0.6–2.9); *acr*B: 1.75 (95% CI: 1.27–2.94)] of the susceptible strains (Figure 2). However, the difference in the relative expression of the *tol*C gene was statistically non-significant (*p* = 0.4). In total. 9 and 13 MDR strains over expressed *acr*A and *acr*B, respectively. In contrast, the over expression of *tol*C was observed in only two isolates.

## 3. Discussion

In total, ~13% of the *E. coli* isolates were confirmed as ESBL producers and about 17.6% as ACBL producers in this study. A number of studies have linked the use of high-generation cephalosporins to such increasing trends of β-lactam resistance in animals, such as the introduction of ceftiofur leading to the spread of *bla*CMY2 [13]. However, such a use in goats, particularly those reared under a semi-intensive system, is quite unlikely because of the sheer cost of medication. Therefore, other possible avenues such as the feed, water and environment, which eventually act as reservoirs of drugs and ARGs, could be alternative sources of infection. A limited passive surveillance by our group conducted in these zones revealed a higher usage of fluoroquinolones in the management of goat diarrhoea.

The increased detection of ESBL and cephalosporinase-mediated resistance in food animals is a cause for concern, as such pathogens may easily sneak into the human food chain and transiently localize in the gut [14,15]. The resistant non-pathogenic flora may also transfer their resistance determinants via transposable elements in the gut or via milk or meat. 

The caprine *E. coli* were frequently resistant to tetracycline, enrofloxacin, cefotaxime and gentamicin mirroring the previous observation in pastured goats [16]. While tetracycline and gentamicin have long been used in veterinary practices, the newer generation of cephalosporins are a recent introduction. Due to their unique structural configurations, both tetracycline and fluoroquinolone with di- and trivalent cations form stable complexes and thereby persist in the environment for longer periods [17]. Similarly, animals treated with these antibiotics may continue to store and periodically shed these compounds or their metabolites, thereby facilitating the spread of resistance genes to other in-contact animals. 

From the correlogram and cluster analysis, it appears that ACBL acts as an important determiner for β-lactam resistance among the caprine isolates (Figure 1). The ACBLs, unlike ESBL isolates, cannot be inhibited by potentiated β-lactamase inhibitors such as clavulanic acid. Therefore, ACBL producers may exhibit resistance to potentiated β-lactam (amoxicillin-clavulanic acid) [18]. 

The higher detection of ACBL-EC (*p* < 0.001) and ESBL-EC in animals with diarrhoea was an interesting observation in this study. ESBL-EC are known to cause difficult-to-treat infections in human and several studies conducted in India pointed out a higher prevalence of ESBLs in tertiary care hospitals and even their spread in the community [9]. While co-morbidities, antibiotic treatments and hospital visits are known risk factors for the colonization of ESBLs in humans, no such risk factor, except AMU linked to intensive farming, has been established in food animals. Interestingly, we failed to extract any significant association between the occurrences of ESBL-EC or ACBL-EC and farming practices. The employed farming practice and the occurrence of diarrhoea appeared to be the significant (*p* < 0.05) contributors to the occurrence of cefotaxime/enrofloxacin resistance among caprine EC isolates. Passive data (not shown) collected from the field revealed that enrofloxacin is one of the commonly prescribed drugs to treat enteric infections in veterinary practice because of its broad-spectrum activity. In our previous studies, we reported that many of the *Enterobacteriaceae* isolates of an animal origin carried both plasmid-mediated quinolone and β-lactam resistance determinants [7,8]. Therefore, the use of enrofloxacin may help provide a congenial selection pressure enabling the bacterial isolates to capture resistance traits for both enrofloxacin and cefotaxime. Further, the role of the environmental spread of resistant bacteria or resistance determinants cannot be ruled out.

The distribution of virulence genes was significantly higher among the isolates from diarrhoeic animals (*iucD*: χ^2^:4.8, *p* = 0.02; *papC*: χ^2^:5.3, *p* = 0.02). However, we could not find any significant association of these virulence factors with the isolates being MDR or ESBL producers. This study showed a significant predominance of uropathogenic virulence factors among the isolates from goats with diarrhoea, although such an association could not be made with their resistance features. Earlier, we detected quite a few isolates with AMR and virulence characteristics among the isolates from poultry in our previous studies [19]. Host-related gut immunity may have a role in determining the colonization of such pathogens.

The exact role of a diarrhoeic condition in the colonization of the resistant flora is still not clear. In a recent study, we also found an increased prevalence of resistant *Enterobacteriaceae* such as ESBL producers in diarrhoeic cattle [8]. In fact, the colonization of *E. coli* in the colon or rectum depends on the presence of extra-intestinal virulence factors such as a P or I type fimbriae-like resident population in the colon [20]. Therefore, these resident populations may have acquired ESBL and other determinants from a transient-resistant carrier through transposable elements [21]. Our previous studies reflecting the co-carriage of ESBL genes and ExPEC virulence by animal *E. coli* isolates are an indicative of such possibility [8,19].

Limited studies are available regarding the characterization of AMR pathogens in goats from the Indian sub-continent to substantiate our findings. A recent study conducted in Assam, India, could not detect any ESBL producers [22]. In contrast, more than 50% of the fecal *E. coli* from healthy and diarrhoeic sheep and goats in Saudi Arabia were MDR, carrying *bla*CTX-M, PMQR and *rmtB* genes [23]. Further, Subramanya et al. [24] also detected ESBL among 51% of the fecal *Enterobacteriaceae* isolates from goats in Nepal. These findings clearly indicate that the prevalence of antibiotic resistance in goats varies widely depending on the sampling source and design, methodology and agro-climatic factors. 

The detection of multiple variants of plasmids in this study is not surprising due to their frequent connection with β-lactamase, carbapenemase and PMQR genes [25,26]. Thirteen (13) isolates harboured IncN plasmids, which are well-established vehicles of resistance genes such as *bla*VIM-1 in humans and *bla*CTX-M-1 in other animals [27]. However, the IncN plasmids detected in this study were among the isolates, which invariably carried *tetA* with or without CTX-M-1, echoing a recent observation that IncN often moves with the tetracycline resistance transposon Tn1721 [28]. In addition, 10 isolates carried IncHI1, which was reported to carry multiple resistance and virulence determinants together [29]. 

The tripartite *acrA–acrB–tolC* system is the major efflux system involved in the expulsion of the cytotoxic molecules such as multiple antibiotics, heavy metals, dyes, detergents and bile salts; consequently, the system is known to play a major role in the multi-drug resistance of the Enterobacteriaceae. Quite similar to the observation of Camp et al. [30], this study revealed an enhanced expression of the *acrAB* system in only up to 30% of the MDR strains. However, the significant elevation in the expression of the *acrAB* gene among the MDR strains in goats could have been due to a prolonged exposure to environmental toxicants, biocides and xenobiotics [31].

This is the first ever study to unravel the hitherto unknown status of AMR in goat farming—a practice employing an animal reared by almost every rural household in the Indian sub-continent. It is apparent that not only the intensive practices but also a common condition such as diarrhoea in the host may increase the likelihood of an isolate having resistant determinants such as ESBL. Further, this study revealed the presence of MDR isolates, which are resistant to even reserved antibiotics such as carbapenem, in goats, thereby revealing a cause for critical public health concern. Given its intricate and multi-sectorial dimensions, this study emphasizes the importance of such epidemiological studies for a more comprehensive understanding of the spread and impact of AMR and calls for the restricted use of antimicrobials to mitigate this burgeoning crisis. 

## 4. Materials and Methods 

### 4.1. Sample Collection and Processing

According to the recent census, West Bengal province has a goat population of around 16 million. Assuming that around 20% of the population may harbour AMR pathogens, the sample size was approximated at 95% confidence interval using open-epi version 3. In total, 244 rectal swab samples were collected in duplicate from March, 2020 to May, 2021 from healthy (170) and diarrhoeic (74) goats reared under intensive (n = 52) and semi-intensive (n = 192) farming conditions from four agro-climatic zones of West Bengal (Supplementary Figure S1). The physiography of the 4 agro-climatic zones varies considerably. Average annual rainfall in all the zones was around 1500 mm except in red and laterite zones (1100–1300 mm). Altogether, the samples were collected from 4 intensive herds and 24 semi-intensive herds. The strength of intensive herd ranged from 40 to ≥100, whereas the strength of semi-intensive herd was around 5 to 30. While most of the semi-intensive farms could not provide any systematic treatment record, the animals from intensive farms received periodic anthelmintics and need-based medical interventions. In general, animals from most of the herds were vaccinated against goat pox and PPR. All the samples were collected aseptically in transport medium and shifted to the laboratory while maintaining cold chain. Samples were inoculated in Mueller Hinton (MH) broth and kept at 37 °C overnight.

### 4.2. Isolation and Confirmation of Pathogens

The samples with visible growths in MH broth were inoculated into MacConkey agar (HiMedia, Mumbai, India). Pink and red-rose colonies were further streaked in Eosin Methylene Blue agar (HiMedia, India) for selective isolation of *E. coli* and were finally confirmed first through standard biochemical test and then by PCR [32]. At least two purified and confirmed colonies per sample were preserved and analysed in order to ascertain randomization and to avoid misinterpretation of the resistance feature.

### 4.3. Antibiotic Resistance Profile of the Caprine E. coli Isolates

Disk diffusion method was employed for susceptibility testing using commercially available discs (BD Life Sciences, NJ, USA): amoxicillin-clavulanic acid (AMC-20/10 µg), cotrimoxazole [COT-25 µg, trimethoprim/sulphamethoxazole-1.25/23.75 µg], aztreonam (ATM-30 µg), ceftiofur (XNL-30 µg), enrofloxacin (ENR-5 µg), cefotaxime (CTX-30 µg), cefpodoxime (CPD-10 µg), chloramphenicol (C-30 µg), tetracycline (TET-30 µg), norfloxacin (NR-10 µg), gentamicin (GEN-30 µg) and imipenem (IPM-10 µg). Isolates with reduced susceptibility to three or more groups of antimicrobials were characterized as MDR [33]. Further, combination disk and cefoxitin-cloxacillin double disk synergy tests were performed for all the confirmed *E. coli* isolates to detect ESBL [34] and AmpC β-lactamase (ACBL) production [35]. *Klebsiella pneumoniae* (ATCC 700603) [ESBL-positive], in-house *E. coli* strains ivkd67 [AmpC β-lactamase positive] and ivkd2 [carbapenemase-positive] and *E. coli* strain ATCC 25922 [negative control] were used as reference. The results of antimicrobial susceptibility test were interpreted as per recent CLSI guidelines using WHO-NET software 5.6.

### 4.4. Clustering of Antibiotics and β-Lactam Resistance Mechanism and Their Correlation

This study was conducted to understand the relationship between all the antibiotics among themselves and with the β-lactam resistance mechanisms (ESBL and ACBL). Accordingly, the resistance or susceptibility profile for each antibiotic and the presence or absence of β-lactam resistance mechanism in the individual caprine isolate investigated in this study were incorporated in binary form for construction of a hierarchical cluster by unweighted pair group method together with arithmetic mean (UPGMA) agglomeration method and a dissimilarity structure produced by Jaccard distance. To ascertain the asymmetric association between each pair of categorical variables (antibiotics), the Goodman and Kruskal tau measures were estimated to produce a square matrix of Goodman and Kruskal measures and the GK-tau-Dataframe was plotted employing Goodman–Kruskal package using R software (version 4.2.1; R Foundation for Statistical Computing).

### 4.5. Development of Logistic Regression Model for Prediction of Resistance Profile of the Caprine Isolates

First, we determined the collinearity among the possible predictors such as age, sex, health status (diarrhoea vs. non-diarrhoea), farming practice (intensive vs. semi-intensive) and the agro-climatic zones. The occurrence of diarrhoea was highest among the goats under 1 year (18%) followed by adolescents (1–2 years; 12%) and adults (more than 2 year: 11%) and the difference was found marginally significant (*p* = 0.07). Therefore, the collinearity between age and other predictors such as diarrhoea could not be ruled out. Further, female goats predominated in most of the herds. Therefore, neither age nor sex were included as explanatory variables in the analysis. The weak or absent correlation among the other predictors or explanatory variables was ascertained by determining Cramér’s V (ranges between 0.02 to 0.18) and corrected contingency coefficient (ranges between 0.02 to 0.16). Finally, logistic regression was performed using general linear model with logit link function to find an association of the resistance profile of caprine isolates against the explanatory variables. The resistance profile of each isolate was incorporated in binary form (susceptible/resistant) after regrouping the intermediate isolates as resistant. For all the resistance characteristics including the antimicrobials, the odds of an isolate being resistant were extrapolated against the health status, rearing condition and zones. As the isolates from the same animals were likely to have similar resistance phenotypes, random effects were incorporated in the model. Finally, the modeling of the log odds of resistance versus susceptible was carried out as a function of host’s health, farming practices and agro-climatic zones. Analysis was performed using R software (version 4.2.1; R Foundation for Statistical Computing).

### 4.6. Identification of Antibiotic Resistance Genes, Virulence Repertoire and Plasmid Incompatibility Types

DNA extracted from phenotypically confirmed MDR *E. coli* (bacterial DNA isolation kit, Zymo Research, USA) was screened for various β-lactamases (*bla*CTX-M, *bla*OXA, *bla*SHV, *bla*TEM, *bla*Amp*C* and *bla*CMY), including carbapenemase (*bla*NDM, *bla*KPC and *bla*OXA-48) genes [8,36]. PCRs were performed to detect quinolone (*qnrA, qnrB, qnrS,* and *aac(6′)-Ib-cr*), tetracycline (*tet*A, *tet*B, *tet*C, *tet*D and *tet*E) and sulfonamide resistance (*sul*1 and *sul*2) genes [7,18]. PCR-based replicon typing was used to determine the diversity of multi-drug resistance plasmids of various incompatibility groups (FIA, FIB, FIC, HI1, HI2, I1-Iγ, L/M, N, P, W, T, A/C, K, B/O, X, Y, F and FIIA) [37]. Only 79 isolates, which included 64 ESBL-positive (MDR: 20, non-MDR: 44) and 15 ESBL-negative but MDR isolates, were subjected to a series of multiplex PCR tests for presence of different diarrhoeagenic factors such as virulence determinants associated with shiga-toxin-producing, enterotoxigenic and uropathogenic *E. coli* [19]. Of them, 27 belonged to diarrhoeic animals and 52 to healthy goats.

### 4.7. Phenotypic Investigation of Efflux Pump Activity in the MDR Isolates

To investigate the role of efflux pumps, all the MDR isolates were evaluated by using meropenem-carbonyl-cyanide-m-chlorophenylhydrazone (MEM-CCCP) disc synergy test [38]. Simultaneously, minimum inhibitory concentrations of ciprofloxacin were determined with or without adding efflux pump inhibitors (CCCP and phenylalanine arginine β-naphthylamide) at sub-inhibitory concentrations for understanding any role of efflux pumps in fluoroquinolone resistance [39]

### 4.8. Transcriptional Expression of acrA, acrB and tolC Genes

In order to investigate the role of *AcrAB* efflux system, all the MDR *E. coli* and 10 pan-susceptible *E. coli* strains (isolated in this study) were screened through quantitative PCRs (qPCRs) (Biorad-CFX96^TM^, Singapore) via iQ SYBR Green Supermix kit (Biorad, Hercules, CA, USA) using primers and PCR conditions described elsewhere [40]. The housekeeping gene (*rps*L) was used as internal control to calculate the relative expression. While the genomic DNA of *E. coli* (ATCC 25922) was kept as positive control, their cDNA was used as calibrator. The difference in relative expression (for *acrA, acrB* and *tolC*) between the MDR and pan-susceptible *E. coli* strains was determined by non-parametric Mann–Whitney U test. The mean of the relative expression of each gene in the 10 pan-susceptible isolates was calculated and the cut-off value for over expression was fixed at two standard deviations (SD) above the corresponding means (Mean + 2 × SD). All the analyses were conducted in R software (version 4.2.1; R Foundation for Statistical Computing).

## Figures and Tables

**Table 1:** Different risk factors contributing to the resistance characteristics of caprine *Escherichia coli* isolate.

Resistance Characteristics	Risk Factors	Proportion (%) with 95% CI	Odds Ratio with 95% CI	Significance
ESBL positivity	Farming practice			
	*Intensive* *	11.8 (8.8–14.8)	1.1 (0.6–2.0)	ns
	*Semi-intensive* *	14.6 (9.5–19.9)		
	Health condition			
	*With diarrhoea* **	14.6 (9.5–20)	1.4 (0.8–2.4)	ns
	*Without diarrhoea* *	10.9 (8–13.9)		
	Agro-climatic zones			
	*Coastal saline zone* *	12.2 (7.7–17.2)	1.1 (0.6–2.2)	ns
	*Red and laterite zone* *	13.0 (8.2–18.4)	0.8 (0.4–1.8)	
	*New alluvial zone* *	10.3 (6–16)	1.0 (0.5–2.1)	
	*Old alluvial zone* *	12.2 (7–18)		
ACBL positivity	Farming practice			
	*Intensive* *	19.1 (12.7–25.8)	1.3 (0.7–2.1)	ns
	*Semi-intensive* *	15.7 (12.5–19.3)		
	Health condition			
	*With diarrhoea* **	22.9 (16.6–29.3)	1.9 (1.2–3.0)	0.001
	*Without diarrhoea* *	13.8 (10.6–17.3)		
	Agro-climatic zones			
	*Coastal saline zone* *	17.3 (12.2–23.5)		
	*Red and laterite zone* *	16.4 (11.0–22.4)	1.0 (0.5–1.8)	ns
	*New alluvial zone* *	15.4 (9.4–1.6)	0.9 (0.5–1.7)	
	*Old alluvial zone* *	16.5 (10.4–23.2)	0.9 (0.5–1.8)	
Ampicillin resistance	Farming practice			
	*Intensive* **	29.4 (22.2–37.9)	1.4 (0.9–2.2)	ns
	*Semi-intensive* **	22.6 (18.6–26.7)		
	Health condition			
	*With diarrhoea* **	26.8 (20.4–34.0)	1.2 (0.8–1.9)	ns
	*Without diarrhoea* **	23.1 (19.1–27.5)		
	Agro-climatic zones			
	*Coastal saline zone* **	25.0 (18.6–31.9)		
	*Red and laterite zone* **	24.7 (18.5–32.1)	1.0 (0.6–1.7)	ns
	*New alluvial zone* **	21.4 (14.5–28.8)	0.9 (0.5–1.5)	
	*Old alluvial zone* **	25.2 (18.3–33.7)	1.1 (0.6–1.9)	
Amoxycillin-clavulanic acid resistance	Farming practice			
	*Intensive* *	15.1(9.5–1.3)	1.3 (0.7–2.4)	ns
	*Semi-intensive* *	11.5 (8.6–4.5)		
	Health condition			
	*With diarrhoea* *	14.0 (9.6–9.7)	1.2 (0.7–2.2)	ns
	*Without diarrhoea* *	11.7 (8.8–14.9)		
	Agro-climatic zones			
	*Coastal saline zone* *	12.8 (8.3–18.1)		
	*Red and laterite zone* *	13.7 (8.9–19.4)	1.1 (0.6–2.2)	ns
	*New alluvial zone* *	10.3 (6.0–16.0)	0.8 (0.4–1.8)	
	*Old alluvial zone* *	12.2 (7.0–18.0)	1.0 (0.5–2.0)	
Cefotaxime resistance	Farming practice			
	*Intensive* **	28.6 (21.4–37.0)	1.50 (0.97–2.31)	0.05
	*Semi-intensive* **	21.1 (17.4–25.2)		
	Health condition			
	*With diarrhoea* **	28.0 (21.7–35.5)	1.50 (0.94–2.38)	0.05
	*Without diarrhoea* **	20.7 (16.7–24.8)		
	Agro-climatic zones			
	*Coastal saline zone* **	25.0 (18.6–31.9)		
	*Red and laterite zone* **	20.6 (14.4 -27.0)	0.81 (0.47–1.40)	ns
	*New alluvial zone* **	22.2 (15.4 -29.9)	0.92 (0.51–1.64)	
	*Old alluvial zone* **	23.5 (16.5–31.5)	0.95 (0.53–1.69)	
Amikacin resistance	Farming practice			
	*Intensive* **	22.2 (15.9–29.9)	1.05 (0.63–1.70)	ns
	*Semi-intensive* **	21.0 (17.0- 25.1)		
	Health condition	21.1 (17.4–25.2)		
	*With diarrhoea* **	22.3 (16.6–29.2)	1.09 (0.69–1.71)	ns
	*Without diarrhoea* **	21.0 (17.0- 25.1)		
	Agro-climatic zones			
	*Coastal saline zone* **	22.4 (16.7–29.4)		
	*Red and laterite zone* **	21.9 (15.8–28.8)	0.98 (0.56–1.69)	ns
	*New alluvial zone* **	20.5 (13.7–27.7)	0.90 (0.49–1.62)	
	*Old alluvial zone* **	20.0 (13.9–27.8)	0.86 (0.47–1.57)	
Tetracycline resistance	Farming practice			
	*Intensive* **	26.98 (19.84–35.04)	1.4 (0.8–2.1)	ns
	*Semi-intensive* **	22.06 (18.14–26.09)		
	Health condition			
	*With diarrhoea* **	28.66(22.29–36.26)	1.5 (1.0–2.3)	ns
	*Without diarrhoea* **	20.95(16.98–25.06)		
	Agro-climatic zones			
	*Coastal saline zon e* **	22.44(16.67–29.36)		
	*Red and laterite zone* **	22.6(16.44–29.61)	1.0 (0.6–1.8)	ns
	*New alluvial zone* **	23.93(17.09–32.07)	1.1 (0.6–2.0)	
	*Old alluvial zone* **	24.35 (17.39–32.6)	1.1 (0.6–2.0)	
Enrofloxacin resistance	Farming practice			
	*Intensive* **	31.8(23.8–39.9)	1.7 (1.0–2.6)	0.01
	*Semi-intensive* **	22.3 (18.4–26.4)		
	Health condition			
	*With diarrhoea* **	31.2 (24.2–38.6)	1.6 (1.1–2.5)	0.01
	*Without diarrhoea* **	21.8 (17.8–26.0)		
	Agro-climatic zones			
	*Coastal saline zone* **	25.0 (18.6–31.9)		
	*Red and laterite zone* **	24.0 (17.8–31.3)	1.0 (0.6–1.7)	ns
	*New alluvial zone* **	22.2 (15.4–29.9)	0.9 (0.5–1.7)	
	*Old alluvial zone* **	27.0 (19.1–35.0)	1.2 (0.7–2.0)	
Cotrimoxazole resistance	Farming practice			
	*Intensive* **	14.29 (8.73–20.12)	1.3 (0.7–2.3)	ns
	*Semi-intensive* **	11.52 (8.58–14.46)		
	Health condition			
	*With diarrhoea* **	13.38 (8.92–18.88)	1.2 (0.7–2.1)	ns
	*Without diarrhoea* **	11.67 (8.75–14.92)		
	Agro-climatic zones			
	*Coastal saline zone* **	13.46 (8.97–19)		
	*Red and laterite zone* **	11.64 (7.53–17.12)	0.9 (0.4–1.7)	ns
	*New alluvial zone* **	12.82 (7.69–18.9)	1.0 (0.5–2.0)	
	*Old alluvial zone* **	10.43 (6.09–16.24)	0.8 (0.3–1.6)	
Imipenem resistance	Farming practice			
	*Intensive* *	12.7 (7.94–18.7)	1.6 (0.8–2.9)	ns
	*Semi-intensive*	8.82 (6.37–11.54)		
	Health condition			
	*With diarrhoea* *	11.46 (7.01–16.24)	1.3 (0.7–2.4)	ns
	*Without diarrhoea*	9.02 (6.37–11.79)		
	Agro-climatic zones			
	*Coastal saline zone* *	10.26 (6.41–15.16)		
	*Red and laterite zone*	8.22 (4.79–12.83)	0.8 (0.4–1.8)	ns
	*New alluvial zone* *	11.97 (6.84–17.66)	1.3 (0.6–2.8)	
	*Old alluvial zone*	8.7 (4.35–13.6)	0.9 (0.4–2.0)	

* Resistance level—moderate; ** resistance level—high; for others, resistance levels were low (as per EFSA guideline).

**Table 2:** Antibiotic resistance, β-lactamase production, efflux pump-mediated carbapenem and fluoroquinolone resistance and plasmid profiling of multi-drug resistant *Escherichia coli* isolates of caprine origin in 4 different agro-climatic regions of West Bengal, India.

Isolates	Resistance Genotype	ESBL	AmpC-βL	MBL	BF	MEM-CCCP	EP (CIP)	Plasmid Replicon
CaEC1	*bla*CTXM-1-*qnr*B-*aac(6′)-Ib-cr*-*tet*A	P	N	N	MP	N	N	HI1, FIA, FrepB
CaEC2	*bla*CTXM-1-*qnr*B-*tet*A	P	P	N	SP	N	P	FIA, I1, FrepB
CaEC3	*bla*CTXM-1-*qnr*S-*tet*A	P	P	N	SP	P	N	FIA, I1, FrepB
CaEC4	*bla*CTXM-1-*bla*SHV12-*qnr*B-*tet*A	P	P	N	MP	N	N	FIA, I1, FrepB
CaEC5	*bla*AmpC-*tet*A	N	P	N	SP	N	P	N
CaEC6	*bla*CTXM-1-*qnr*B	P	N	N	SP	N	N	I1, FrepB
CaEC8	*bla*CTXM-1-*qnr*B-*aac(6’)-Ib-cr*-*tet*A	P	P	N	SP	N	P	HI1, FIB, FrepB
CaEC9	*bla*AmpC-*bla*CITM	N	N	N	WP	N	N	HI1
CaEC10	*bla*AmpC-*qnr*S-*tet*A	N	P	N	MP	P	N	N, HI1
CaEC12	*bla*CTXM-1-*qnr*S-*aac(6’)-Ib-cr*-*tet*A	P	P	N	MP	N	N	FIA, N, FrepB
CaEC13	*bla*CTXM-1-*bla*SHV12-*qnr*B-*tet*A	P	P	N	MP	N	N	FIA, FIB, FrepB
CaEC15	*bla*CTXM-1-*qnr*S-*tet*B	P	P	N	MP	N	N	FIA, FIC
CaEC16	*bla*CTXM1-*tet*A	P	N	N	MP	N	N	FIB, N
CaEC18	*bla*CTXM-1	P	P	N	MP	N	N	FIA
CaEC19	*bla*CTXM-1-*bla*SHV12-*qnr*B-*tet*A	N	P	N	MP	N	P	FIA, I1, FrepB, N
CaEC20	*bla*CTXM-1-*bla*SHV12-*qnr*S	P	P	N	WP	N	P	FIA, FIB
CaEC22	*bla*AmpC-*tet*A	N	P	N	WP	N	P	N
CaEC23	*bla*AmpC-*bla*CITM-*tet*B	P	P	N	MP	N	N	HI1
CaEC24	*bla*CTXM1-*tet*A	P	P	N	WP	N	N	FIB, N, FrepB
CaEC25	*bla*CTXM1-*tet*A	P	P	N	WP	N	N	FIB, N, FrepB
CaEC26	*bla*AmpC-*bla*CITM-*tet*B	N	N	N	SP	P	N	HI1, FIC
CaEC28	*bla*AmpC-*tet*A	N	P	N	MP	N	N	N
CaEC29	*bla*AmpC-*tet*A-*qnr*S	N	P	N	MP	N	N	FIA, N
CaEC30	*bla*CTXM1-*tet*B-*qnr*B-CMY-6	P	P	N	WP	N	N	FIA, FIB, HI1, FrepB
CaEC31	*bla*CTXM-1-*bla*SHV12-*qnr*B-*tet*A	N	P	N	WP	N	P	I1, FIA, FrepB
CaEC32	*bla*CTXM-1- *bla*SHV12- *bla*AmpC	N	P	N	MP	N	N	FIB, FepB
CaEC33	*bla*NDM-5-*bla*TEM-*sul*1	P	N	P	WP	N	N	FIB, A/C, FrepB
CaEC34	*bla*CTXM-1-*bla*SHV12-ampC	P	P	N	SP	N	N	FIA, FrepB
CaEC36	*bla*TEM-*sul*1	N	N	N	WP	N	N	FIC, FrepB
CaEC37	*qnr*B-*qnr*S-*sul*1	N	N	N	MP	N	N	I1, HI1
CaEC38	*bla*AmpC	N	P	N	MP	N	N	Unknown
CaEC40	*bla*CTXM-1-*tet*A	P	N	N	WP	N	P	FIB, FrepB
CaEC41	*bla*CTXM-1-*bla*TEM-ampC	P	P	N	SP	N	P	I1, HI1, FrepB
CaEC42	*bla*NDM-5	N	N	P	MP	N	N	A/C, FrepB
CaEC44	*bla*AmpC-*sul*1-*tet*A	N	P	N	MP	N	P	HI1, N

ESBL—extended spectrum β-lactamases; AmpC-βL—AmpC β-lactamases; BF—biofilm; MEM-CCCP—meropenem-carbonyl-cyanide-m-chlorophenylhydrazone; EP (CIP)—efflux pump activity tested with ciprofloxacin; MBL—metallo-β-lactamases.

## Data Availability

The data used to support the findings of this study are available. Further inquiries can be directed to the corresponding authors.

## Supplementary Materials

The following supporting information can be downloaded at: https://www.mdpi.com/article/10.3390/antibiotics11101344/s1, Figure S1: Conceptual framework and sampling plan of the study carried out on *E. coli* isolates in black Bengal goats covering four agro-climatic zones of West Bengal, India. The graphics for this figure were designed in Inkscape. Figure S2: Proportion of the rectal *Escherichia coli* (n: 488) resistant to different antibiotics with 95% CI.
