# Supplementary material for: Antimicrobial Resistance Pattern, Clustering Mechanisms and Correlation Matrix of Drug-Resistant Escherichia coli in Black Bengal Goats in West Bengal, India
Source: Antibiotics (Basel). 2022 Oct 1;11(10):1344. doi: 10.3390/antibiotics11101344 (PMC9598321; doi:10.3390/antibiotics11101344)
Supplement: Supplementary file 1 [file antibiotics-11-01344-s001.zip › antibiotics-1940131-supplementary.pdf]

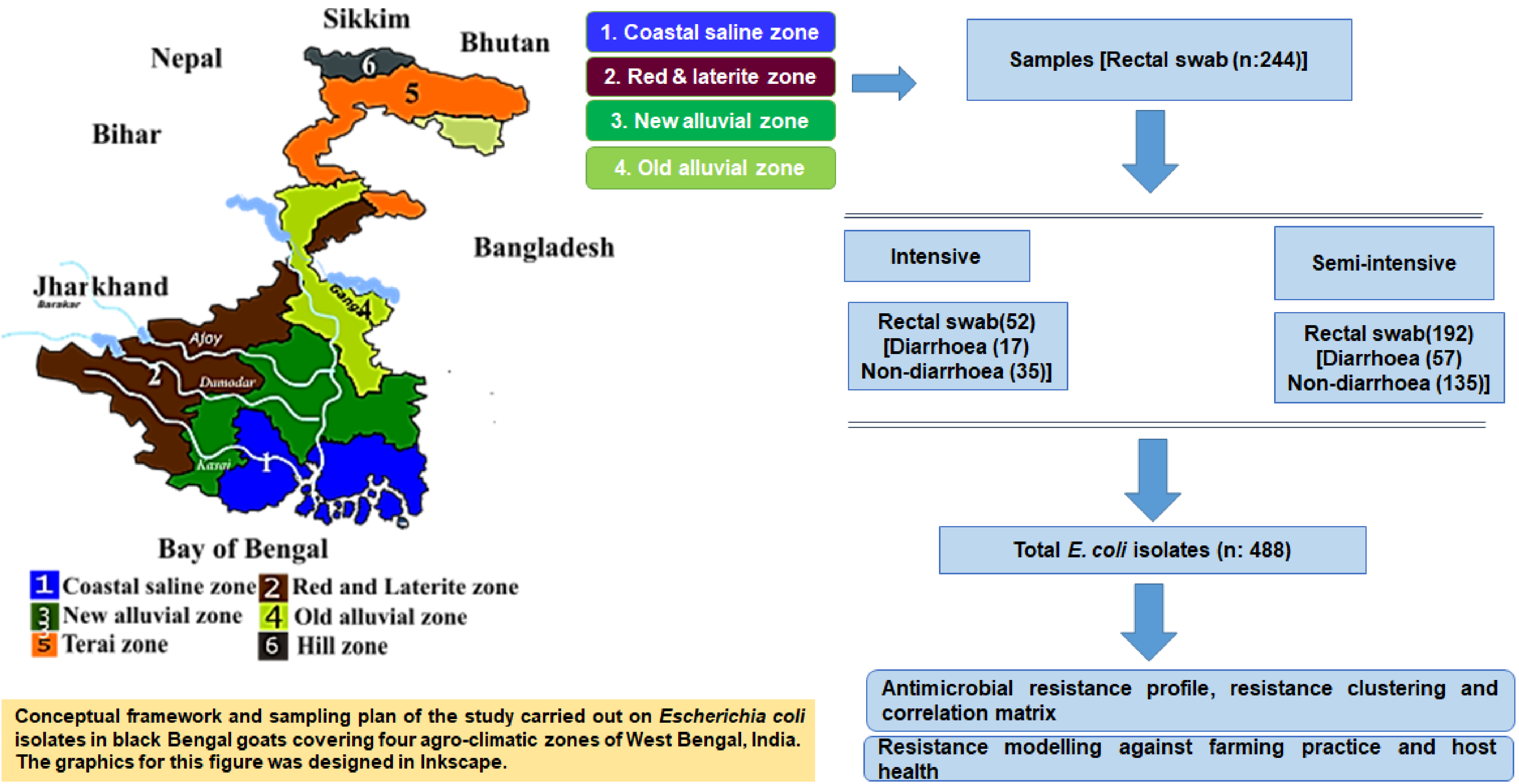

Figure S1: Conceptual framework and sampling plan of the study carried out on *E. coli* isolates in black Bengal goats covering four agro-climatic zones of West Bengal, India. The graphics for this figure was designed in Inkscape.

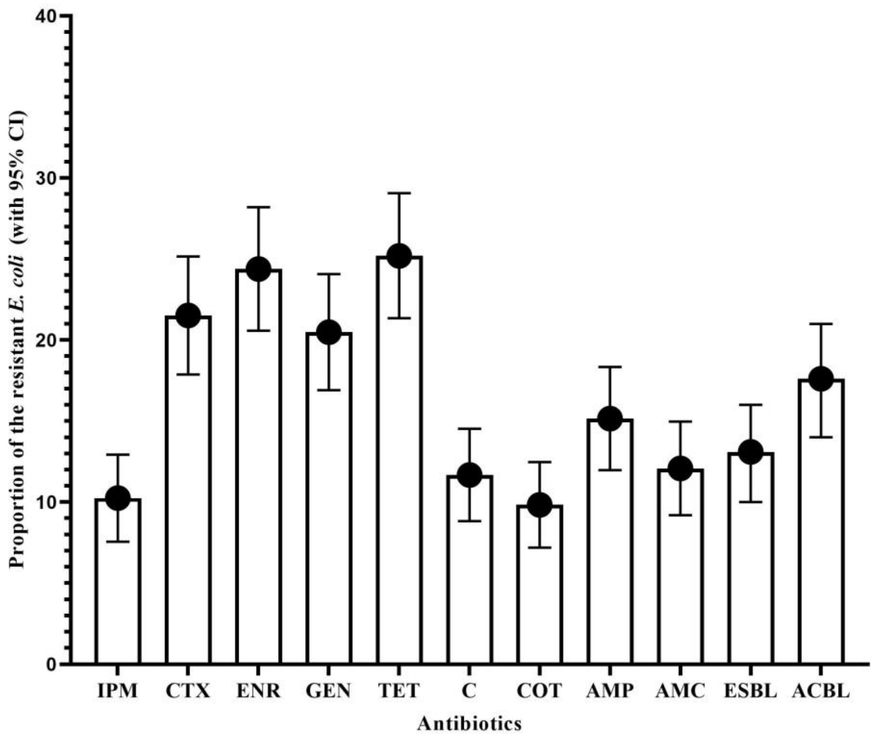

IPM: imipenem, CTX: cefotaxime, ENR: enrofloxacin, GEN: gentamicin, TET: tetracycline, C: chloramphenicol, COT: cotrimoxazole, AMP: ampicillin, AMC: amoxyclav, ESBL: extended spectrum β-lactamase, ACBL: ampC-β-lactamase

Figure S2: Proportion of the rectal *E. coli* (n: 488) resistant to different antibiotics with 95% CI.
